# A Common Polymorphism in the Promoter Region of the TNFSF4 Gene Is Associated with Lower Allele-Specific Expression and Risk of Myocardial Infarction

**DOI:** 10.1371/journal.pone.0017652

**Published:** 2011-03-18

**Authors:** Massimiliano Ria, Jacob Lagercrantz, Ann Samnegård, Susanna Boquist, Anders Hamsten, Per Eriksson

**Affiliations:** 1 Atherosclerosis Research Unit, Center for Molecular Medicine, Department of Medicine, Karolinska University Hospital Solna, Stockholm, Sweden; 2 Division of Cardiovascular Medicine, Department of Clinical Sciences, Danderyd Hospital, Karolinska Institutet, Stockholm, Sweden; Vrije Universiteit Medical Center and Center for Neurogenomics and Cognitive Research, Netherlands

## Abstract

**Background:**

The TNFSF4/TNFRSF4 system, along with several other receptor-ligand pairs, is involved in the recruitment and activation of T-cells and is therefore tentatively implicated in atherosclerosis and acute coronary syndromes. We have previously shown that genetic variants in *TNFSF4* are associated with myocardial infarction (MI) in women. This prompted functional studies of *TNFSF4* expression.

**Methods and Results:**

Based on a screening of the *TNFSF4* genomic region, a promoter polymorphism (rs45454293) and a haplotype were identified, conceivably involved in gene regulation. The rs45454293T-allele, in agreement with the linked rs3850641G-allele, proved to be associated with increased risk of MI in women. Haplotype-specific chromatin immunoprecipitation of activated polymerase II, as a measure of transcriptional activity *in vivo*, suggested that the haplotype including the rs45454293 and rs3850641 polymorphisms is functionally important, the rs45454293T- and rs3850641G-alleles being associated with lower transcriptional activity in cells heterozygous for both polymorphisms. The functional role of rs45454293 on transcriptional levels of *TNFSF4* was clarified by luciferase reporter assays, where the rs45454293T-allele decreased gene expression when compared with the rs45454293C-allele, while the rs3850641 SNP did not have any effect on *TNFSF4* promoter activity. Electromobility shift assay showed that the rs45454293 polymorphism, but not rs3850641, affects the binding of nuclear factors, thus suggesting that the lower transcriptional activity is attributed to binding of one or more transcriptional repressor(s) to the T-allele.

**Conclusions:**

Our data indicate that the *TNFSF4* rs45454293T-allele is associated with lower *TNFSF4* expression and increased risk of MI.

## Introduction

TNFSF4 (also designated OX40L, gp34 and CD134L, GenBank accession no. NM_003326), the ligand of the TNFRSF4 (OX40 receptor), is a member of the tumor necrosis factor (TNF) superfamily. TNFSF4 is a T-cell activator that seems to promote the survival (and perhaps prolong the immune response) of CD4+ T-cells at sites of inflammation [1]. T-cells are indicated to have an essential role in the development of atherosclerosis [2]. Activated CD4+ and CD8+ T-cells, B cells and vascular endothelial cells have been reported to express TNFSF4 [3].

Based on a mouse atherosclerosis model [4] to positionally identify potential human candidate genes, we have provided evidence that genetic variation in the *TNFSF4* gene contributes to the risk of developing myocardial infarction (MI) [5]. A single nucleotide polymorphism (SNP) in the first intron of *TNFSF4* (rs3850641) and haplotypes including this SNP were found to be associated with risk of MI in women in two independent populations and with angiographically assessed severity of CAD. *TNFSF4* haplotypes were also associated with variation in some risk factors for CAD; carriers of a specific haplotype had significantly higher plasma concentrations of HDL cholesterol and serum amyloid A (SAA) than did carriers of other haplotypes. Furthermore, gene targeting of *Tnfsf4* in mice showed that deficient animals had significantly smaller atherosclerotic lesions and higher levels of plasma total cholesterol and HDL cholesterol than controls, while mice over-expressing *Tnfsf4* had significantly larger atherosclerotic lesions when compared to controls, indicating that *Tnfsf4* is a gene influencing atherosclerosis susceptibility in mice. Further support for a role of *TNFSF4* in atherosclerosis was obtained in a study where blockage of the TNFSF4/TNFRSF4 interaction reduced lesion formation in low-density lipoprotein (LDL) receptor-deficient mice [6]. In contrast, a German case-control study failed to replicate the association between *TNFSF4* and MI [7].

The functional polymorphism responsible for the association between *TNFSF4* and risk of MI has not been defined, nor is it known whether high or low expression of *TNFSF4* is associated with MI in man. Since the observed association with MI [5] involved a haplotype spanning regions of the gene located both upstream and downstream of the transcription start site, the functional variant(s) could be located anywhere along the stretch of DNA defined by this haplotype. However, due to its location, the rs3850641 polymorphism in intron 1 is unlikely to be the functional variant. Nevertheless, interaction between the rs3850641 polymorphism and a putative enhancer binding site located in intron 1 cannot be excluded, but a functional variant in linkage disequilibrium with rs3850641 and located in the promoter or in other regulatory regions is a more plausible explanation.

In an attempt to identify the functional SNP(s) or a minimal haplotype suitable for further functional studies, we performed a detailed screening of the *TNFSF4* genomic region. New genetic variants were subsequently examined in one of the clinical studies previously used to establish the association between *TNFSF4* and MI. A promoter polymorphism was identified which is in linkage disequilibrium with the rs3850641 SNP and associated with MI in women. Functional analyses of the transcriptional activity of these genetic variants were then performed both *in vivo*, using haplotype-specific chromatin immunoprecipitation of activated polymerase II (haploChIP assay) [8], and *in vitro* with assays of luciferase reporter gene expression; electromobility shift assay (EMSA) was employed to analyze allele-specific binding of transcription factors *in vitro*.

## Methods

### Human subjects

The biobank and database of the Stockholm Coronary Atherosclerosis Risk Factor (SCARF) study were used for the genotype-phenotype association studies. DNA samples from a total of 387 individuals with MI before age 60 and 387 age- and sex-matched population-based control subjects were examined in the present study. Genotyping was successfully performed in 376 patients with MI and 385 controls, while complete data were obtained from 359 patients and 382 controls. Recruitment procedures, inclusion and exclusion criteria, investigation program and basic clinical characteristics have been described in detail [9]. All subjects gave informed written consent to their participation in the study, the protocol of which had been approved by the ethics committee of the Karolinska University Hospital.

### Sequencing, SNP validation and genotyping

Polymorphisms detected *in silico* were validated by sequencing genomic DNA from whole blood of 20 healthy subjects. Parts of the regulatory region from 2449 bp upstream to 374 bp downstream of the *TNFSF4* transcription start site (2823 bp in total) and parts of intron 1 were PCR amplified and sequenced using a standard cycle-sequencing system (PTC-225, MJ Research, Waltham, MA, USA). Primer sequences are provided in Tables S1 and S2.

Genotyping was performed using TaqMan® assays (Assay-by-Design, Applied Biosystems, Foster City, CA, USA). Post-PCR allelic discrimination was carried out measuring allele-specific fluorescence on the ABI Prism® 7000 Sequence Detection System (Applied Biosystems, Foster City, CA, USA) using the Sequence Detection System software version 1.01.

### Cell culture

THP-1 [10] and U937 [11] cells were grown in RPMI-1640 supplemented with 10% foetal bovine serum (Gibco BRL, Paisley, UK), 1 mM sodium pyruvate, 0.05 mM 2-mercaptoethanol, penicillin (100 U/ml), and streptomycin (100 µg/ml) in humidified air at 37°C and 5% CO2.

For the haploChIP analyses, nine established Epstein-Barr virus (EBV)-transformed human B cell lines [12] (a kind gift from Prof. Anna Wedell, Center for Molecular Medicine, Karolinska Institutet, Stockholm, Sweden) were maintained at 2x106 cells per ml in RPMI 1640 medium with GlutaMAX™ I (Gibco BRL, Paisley, UK) supplemented with 10% heat inactivated FBS, penicillin (100 U/ml), and streptomycin (100 µg/ml). To genotype these cells, DNA extraction was performed using Genomic-tip 100/G kit (Qiagen Nordic, Hilden, Germany) according to the manufacturer's protocol; yield and efficiency of extraction were measured by making quantitative spectrophotometric absorbance readings at 260 nm.

For the luciferase assay HEK293T cells (obtained from ATCC) were grown in Dulbecco's Modified Eagle's medium supplemented with 10% FBS, 2mM glutamine, penicillin (100 U/ml), and streptomycin (100 µg/ml).

### Semi-quantitative reverse transcription polymerase chain reaction (RT-PCR)

Total RNA was prepared with RNeasy kit (Qiagen Nordic, Hilden, Germany) and reverse-transcribed into cDNA by using oligo dT primers and Superscript II (Invitrogen, Life Technologies, Carlsbad, CA, USA) according to the manufacturer's instructions. Real-time RT-PCR was performed on ABI Prism Sequence Detection System 7000 (Applied Biosystems, Foster City, CA, USA) and the results were normalized to the house keeping gene RPLP0. cDNA (3 µL) was amplified by real-time PCR with 1xTaqMan Universal PCR Master Mix and Assay-on-Demand Hs00967195_m1 (Applied Biosystems, Foster City, CA, USA) according to the manufacturer's instructions. Each sample was analyzed in duplicate under the following conditions: 2 min at 50°C, 10 min at 95°C, 0.15 min at 95°C, and 1 min at 60°C. The PCR amplification was related to a standard curve. Reactions were performed in MicroAmp Optical 96-Well Reaction Plates (Applied Biosystems, Foster City, CA, USA). The following primers and probe were used for the amplification of the RPLP0 gene: RPLP0-F-5′-CCA TTC TAT CAT CAA CGG GTA CAA-3′; RPLP0-R-5′-AGC AAG TGG GAA GGT GTA ATC C-3′and probe RPLP0-5′-TCT CCA CAG ACA AGG CCA GGA CTC GT-3′.

### HaploChIP analyses

The chromatin immunoprecipitation assay was performed as described [8] with some modifications. Preparation of chromatin and immunoprecipitation were carried out using the ChIP-IT™ kit (Active Motif, Carlsbad, CA) according to the manufacturer's instructions, with some modifications. A total of 1×10^8^ cells grown in suspension culture were harvested by centrifugation and fixed using volumes adjusted to the amount of cells; all the incubation steps were followed by 5 min centrifugation. Nuclei containing pellets were resuspended in half of the original volume to increase chromatin concentration and sheared using a Branson 450 Sonifier with 12 pulses of 20 sec each (settings: output 4, cycle 85, time seconds) to an optimal length of 1500 bp. Samples were cooled on ice for 30 sec between pulses. For chromatin immunoprecipitation, specific antibodies against phosphorylated serine residues of the c-terminal domain (CTD) of RNA polymerase II (Pol II) (Ser5, MMS-134R clone H14; Biosite AB, Täby, Sweden) were used. An antibody against total Pol II (N-20; sc-899; Santa Cruz Biotechology) was used as a positive control, while anti-SV40 T Antigen pAb101 (sc-147; Santa Cruz Biotechology) was used as mock antibody control. Recovery of antibody bound protein/DNA complexes, washing, collection of immunoprecipitated DNA, reversion of cross-links and DNA purification were performed according to the manufacturer's instructions. Allele-specific loading of Pol II was evaluated by Pyrosequencing™ as described [13]. Briefly, DNA was amplified using primer pairs with one biotinylated 5' end (Table S3) [14]. The amplification products were captured on streptavidin coated sepharose beads (Amersham Biosciences AB, Uppsala, Sweden), denatured and then washed. Pyrosequencing primer was added and analyzed as described [15]. Pyrosequencing was performed using the 5×96 PSQ™ 96MA Pyro Gold Reagents. To quantify the two alleles, measurement of the area under the peak corresponding to each allele was performed using the PSQ™96MA SNP software. Results for each polymorphism were expressed as ratio between the two alleles and normalized to the starting material which was set to 1 to reflect the condition of a heterozygous genomic DNA.

### Luciferase assay

The genomic region upstream of the *TNFSF4* transcription start site (TSS) (from nt -1392 to -6) was amplified with primers 5′-TGTTCTCCTAATGCAAGGCATA and 5′-CAATCTGGGTAGAGGGAAGAT from individuals homozygous for either one or the other allelic variant and cloned into pCRII (Invitrogen, Life Technologies, Carlsbad, CA, USA); this construct was subsequently digested with *KpnI* and *XhoI* to generate a fragment spanning the promoter region from nt -973 to -6 (including the rs45454293 SNP) and transferred to pGL3-Basic expression vector (Promega). Double-stranded DNA fragments (nt 476-492 from TSS) harbouring intron 1 rs3850641 SNP flanked by *KpnI* and *XhoI* restriction sites were generated by annealing of oligonucleotides carrying either one or the other allelic variant (5′-GACT*CTCGAG*CTATCACAATGGGTAGA*GGTACC*GACT). The polymorphic site is underlined while the restrictions sites are in italics. Double-stranded oligonucleotides were cloned into pGL3-Promoter expression vector (Promega) upstream of the SV40 endogenous promoter. Luciferase assay was carried out using the Luciferase Assay System (Promega) according to the manufacturer's protocol. Cells were transfected with 0.1, 0.25 or 0.4 µg of either one of the constructs and 0.25 µg of the -galactosidase CMV-lacZ plasmid as an internal control for transfection efficiency using FuGene6 transfection reagent (Roche Molecular Biochemicals). 48 h after transfection cells were lysed with Reporter Lysis Buffer (Promega), and β-galactosidase and luciferase activity were measured using a Vmax kinetic microplate reader (Molecular Devices, Wokingham, UK) and a dual injection luminometer (Luminoskan Ascent, Labsystem), respectively. Each experiment was independently repeated three times and each sample was studied in duplicate.

### EMSA

The sequences of the double-stranded oligonucleotides used in electrophoretic mobility shift assays (EMSAs) were as follows: rs45454293, 5′-TTTCTTTGAGGTCGTGGCTGGCCTC and rs3850641, 5′-ATTACTATCACAATGGGTAGACCAG. The polymorphic sites are underlined. Nuclear extracts from human THP-1 and U937 cells were prepared according to Alksnis et al. [16]. All buffers were freshly supplemented with 0.7 µg/mL leupeptin, 16.7 µg/mL aprotinin, 0.5 mmol/L PMSF, and 5 mmol/L 2-mercaptoethanol. The protein concentration in the extracts was estimated by the method of Kalb and Bernlohr [17]. Incubation for EMSA was conducted as described [18], [19], and the reaction products were applied to 7% (w/v) polyacrylamide gel (80:1 acrylamide/*N,N*′-methylene-bisacrylamide weight ratio), whereafter electrophoresis was performed in 22.5 mM Tris/22.5 mM boric acid/0.5 mM EDTA buffer for 2 h at 200 V. Non-radioactive competitor DNAs, either identical, of the opposite allelic variant or of non-specific origin, were added to the labelled DNA.

### Bioinformatic sequence evaluation

Genetic variants affecting potential regulatory regions were identified using RAVEN, a web-based application [20]. RAVEN combines phylogenetic footprinting with scanning of all sequence variants for transcription factor binding sites that may be differentially affected by the variation. The Genomatix® package (ElDorado®, Gene2Promoter® and MatInspector®, http://www.genomatix.de/) was used to identify *in silico* potential transcription factor binding sites that were lost or generated due to a SNP in *TNFSF4*. Transcription factor binding sites altered by variants in the *TNFSF4* gene were also evaluated by browsing the TRANSFAC public database 7.0 (http://www.gene-regulation.com/pub/databases.html#transfac) with the web-based softwares TESS (http://www.cbil.upenn.edu/tess) and PATCH (http://www.gene-regulation.com/cgi-bin/pub/programs/patch/bin/patch.cgi).

The ENCODE[21] regions were extracted from the UCSC Genome Browser database. The human NCBI36 (hg18, March 2006) assembly was used (http://genome.ucsc.edu/cgi-bin/hgTracks?db=hg18).

### Statistical analysis

Statistical analyses in SCARF were performed using the StatView software version 5.0 (SAS, Cary, NC, USA). Allele frequencies were estimated by gene counting and tested for deviation from Hardy-Weinberg equilibrium. The chi-square test was used to compare the distribution of genotypes between cases and controls. Differences in continuous variables between groups were tested by analysis of variance (ANOVA) with the Scheffe F-test as a post-hoc test. Pairwise linkage disequilibrium coefficients (D′ and r^2^) for polymorphisms within the *TNFSF4* locus were calculated with the EMLD program developed by Qiqing Huang (http://epi.mdanderson.org/~qhuang/Software/pub.htm) and visualized with the Haploview program (version 3.0) developed by Barret & Maller (http://www.broad.mit.edu/mpg/haploview/) using both our own data and data available from the HapMap Project (http://www.hapmap.org/). Haplotype frequencies were estimated using the PHASE^©^ program (version 2.1) [22].

## Results

### Identification of a novel *TNFSF4* promoter polymorphism associated with risk of MI in women

To identify the physiological variant(s) responsible for the previously reported associations between *TNFSF4* genotype/haplotype and MI [5] and to further define linkage disequilibrium blocks, additional SNPs were selected from public databases and verified by DNA sequencing. Sequencing of 2449 base pairs (bp) upstream and 374 bp downstream of the *TNFSF4* transcription start site (2823 bp in total) in 20 healthy subjects verified the presence of three variants (ID: rs1234315, rs10489266 and rs1234314) previously reported in the Ensembl Human Genome Browser. In addition, a genetic variant previously described in Japanese individuals [23] consisting of a C-to-T substitution located 921 bp upstream of the transcription start site was detected (submitted to and registered in NCBI dbSNP with Reference number rs45454293). In contrast, the rs4531318, rs12027059, rs12045464 and rs12144295 SNPs were not detected. Sequencing of relevant parts of intron 1 confirmed the presence of five SNPs (rs5778749, rs4916314, rs10912564, rs10489267 and rs10912558) while the rs3861951, rs35446169, rs7529929 and rs4113833 SNPs were not detected.

**Figure 1:**
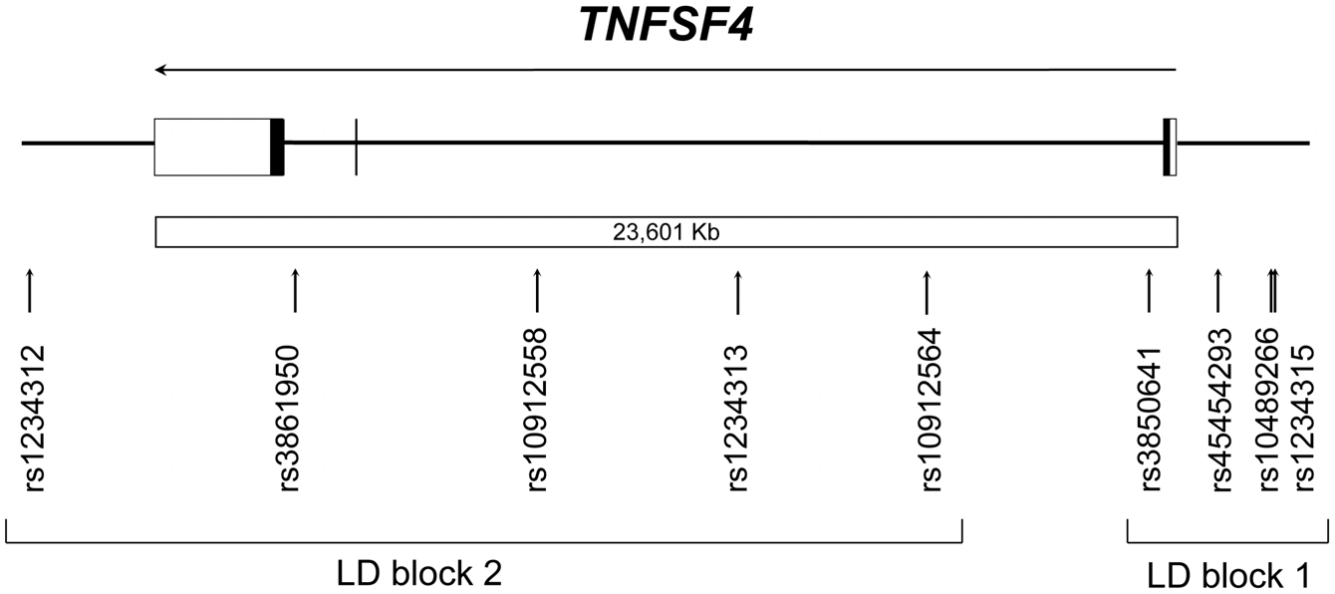
The *TNFSF4* gene as represented in the NCBI database. Vertical arrows denote SNPs tested in human subjects. Horizontal arrow indicates direction of transcription. Exons are depicted by boxes: filled-in regions indicate translated portions of the gene and untranslated regions are depicted by open boxes; introns are indicated as solid lines between boxes. LD: Linkage disequlibrium.

Since one of the aims was to refine linkage disequilibrium blocks described earlier [5], validated alternative SNPs were selected that were positioned in between the five previously genotyped variants [5]. For each set of alternative variants within the same region, the polymorphism having the highest minor allele frequency was considered. Three SNPs (rs10489266, rs10912564 and rs10912558) were selected along with the promoter variant rs45454293 and genotyped in 387 post-infarction patients and 387 age- and sex-matched controls (Fig. 1, Table 1). Of note, the rs1234315, rs3850641, rs1234313, rs3861950 and rs1234312 SNPs have been genotyped previously in the same sample [5].

**Table 1:** Allele frequencies and pairwise linkage disequilibrium coefficients for the SNPs harboured in the *TNFSF4* gene.

		Normalized linkage disequilibrium coefficient r^2^ (|D′|)							
Polymorphism	Minor allele frequencies†	rs1234315	rs10489266	rs45454293	rs3850641	rs10912564	rs1234313	rs10912558	rs3861950
rs1234315*	46.1/43.6								
rs10489266	9.2/8.7	0.12 (1)							
*rs45454293*	7.6/6.1	0.09 (1)	0.17 (1)						
rs3850641*	15.4/12.0	0.19 (0.99)	0.22 (0.83)	*0.70 (0.81)*					
**rs10912564**	32.4/32.6	0.001 (0.01)	0.05 (0.78)	0.02 (0.26)	0.04 (0.53)				
rs1234313*	29.4/32.9	0.06 (0.86)	0.06 (0.93)	0.06 (0.91)	0.73 (0.93)	0.21 (0.98)			
**rs10912558**	20.3/20.3	0.01 (0.21)	0.06 (0.80)	1 (1)	0.02 (0.28)	**0.50 (0.98)**	0.10 (0.94)		
rs3861950*	33.4/30.1	0.003 (0.04)	0.06 (0.83)	0.02 (0.25)	0.04 (0.56)	**0.89 (0.95)**	0.20 (0.98)	**0.47 (0.94)**	
rs1234312*	5.6/5.2	0.02 (0.31)	0.52 (1)	0.001 (0.01)	0.02 (0.31)	0.01 (1)	0.01 (1)	0.007 (1)	0.013 (1)

Results obtained in the combined male and female groups. *Polymorphisms that have been reported previously [5].

† patients/controls. The two haplotype blocks identified across the gene are underlined and polymorphisms within each block having a high value for both D′ and r^2^ are indicated either in italics or bold, respectively.

The genotype distributions of all SNPs tested were found to be in Hardy-Weinberg equilibrium in both cases and controls (Table S4). When evaluated together with the previously genotyped SNPs [5], a high degree of linkage disequilibrium was found between rs1234315, rs10489266, rs45454293 and rs3850641 and between rs10912564, rs1234313, rs10912558, rs3861950 and rs1234312, using the EMLD program (Table 1 and Fig. S1); however when r-squared (r^2^) values were calculated, the two blocks appeared to be smaller, linkage disequilibrium being maintained only between rs45454293 and rs3850641 within the first block, and between rs10912564, rs10912558 and rs3861950 within the second one (Table 1). There was no difference in genotype distribution between all cases and controls for the four new SNPs tested. However, similar to findings for the intron 1 rs3850641 polymorphism [5], the minor T-allele of the rs45454293 substitution was significantly more frequent in female patients than in female controls (C/T allele frequencies: 0.89/0.11 vs 0.96/0.04, n = 262, P = 0.02). Minor allele frequencies encountered in patients and controls are reported in Table 1. Of note, among the four SNPs included in the smaller block spanning the transcription start site, only rs45454293 and rs3850641 showed an association with MI in women. The minor allele frequencies of rs1234315 and rs10489266 (Table 1) suggest that the lack of association with MI, despite strong linkage disequilibrium with the rs45454293 and rs3850641 SNPs, is real rather than due to rarity of these variants. In addition, in the female group the haplotype containing the minor T-allele for rs45454293 and the minor G-allele for rs3850641 was significantly more common in patients than in controls (P = 0.01) (Table 2). Conversely, the complementary CA haplotype carrying both major alleles was more common in controls than in patients (P = 0.02). There were no associations between haplotypes and risk of MI in the combined male and female group (Table S5).

**Table 2:** Distribution of *TNFSF4M* haplotypes in female patients and control subjects (131 individuals/262 alleles).

Haplotype	Controls (%)	Patients (%)	P value	Females/Total (%)*
**00**	120 (88.0)	98 (77.8)	**0.02**	218/1302 (17)
01	7 (5.1)	9 (7.1)	ns	16/120 (13)
10	2 (1.5)	1 (0.8)	ns	3/16 (19)
**11**	7 (5.1)	18 (14.3)	**0.01**	**25/90 (28)**

P values were calculated using the chi-square test for genotype distribution. 0 =  ancestral allele, 1 =  minor allele.

*Proportion of females among carriers of each haplotype in the overall population (Total).

### The rs45454293T and rs3850641G alleles are associated with lower loading of active polymerase II

The haploChIP method was used to analyze allele-specific promoter activity, i.e. the loading status of phosphorylated active Pol II to the *TNFSF4* gene associated with the rs45454293 and rs3850641 polymorphisms was analyzed in cells which were heterozygous for the two markers. The phosphorylated Ser5 residue of the c-terminal domain of Pol II was used as a marker for phosphorylated active Pol II loading, and the relative concentration of phosphorylated Pol II binding to the two alleles was analyzed by pyrosequencing. A panel of nine different human B cell lines transformed with EBV and the monocytic cell line U937 were screened for the rs45454293 and rs3850641 SNPs. Only one B cell line was found to be C/T for rs45454293 and A/G for rs3850641 while the other cell lines were either heterozygous for one SNP or the other (i.e. C/C, A/G or C/T, A/A), or homozygous for both SNPs (C/C, A/A).

**Figure 2:**
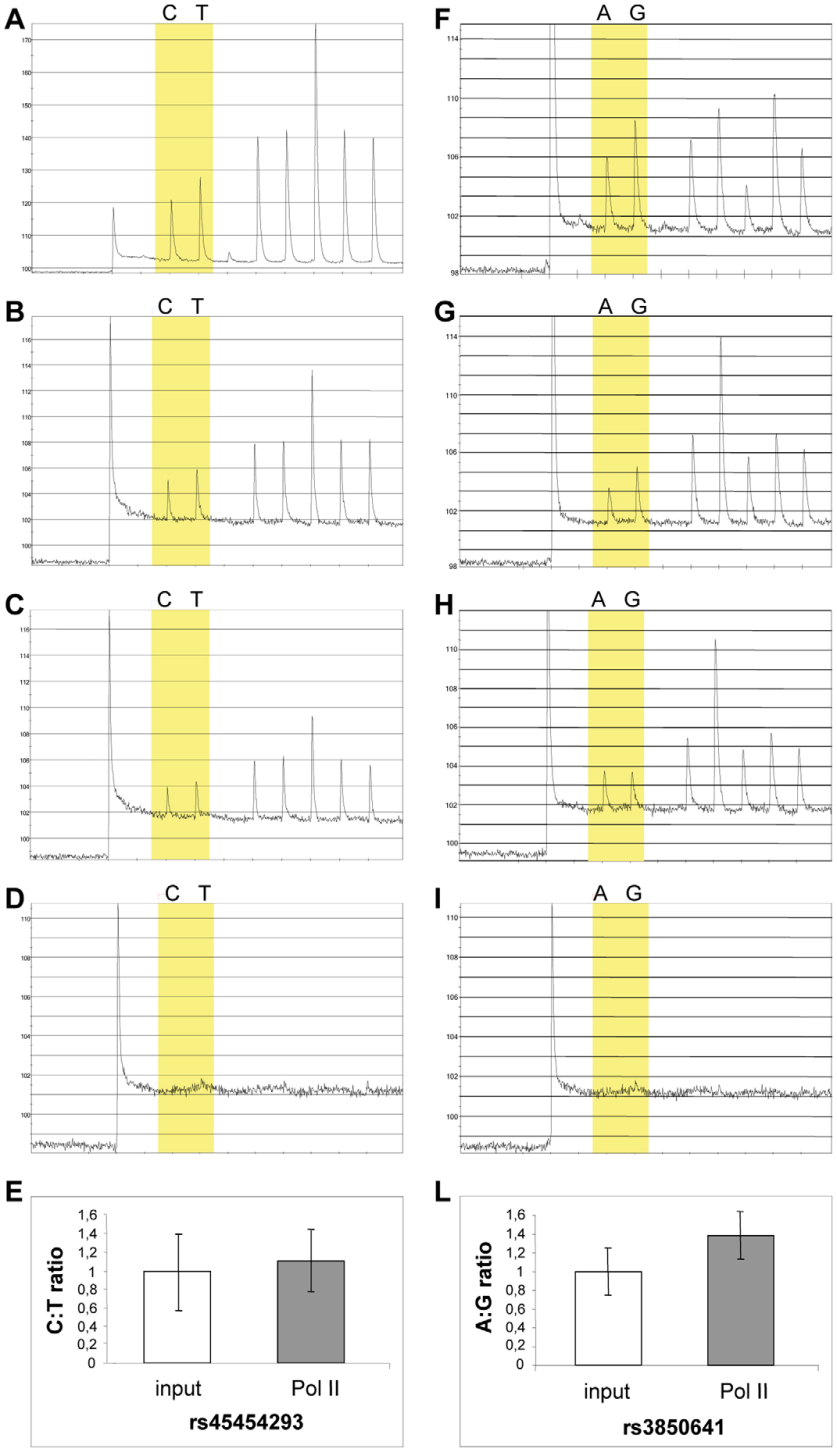
Allele-specific loading of phosphorylated Pol II *in vivo* at rs45454293 (A-D) and rs3850641 (F-I) sites. To quantify the relative levels of abundance of allele-specific fragments, pyrosequencing was used to analyze input chromatin used in ChIP reactions (**A, F**); products of ChIP using specific antibodies to total Pol II as positive control (**B, G**); to phosphorylated serine residues of Pol II CTD (**C, H**); or to SV40 T antigen as mock antibody control (**D, I**). Graphs show input nucleotide sequence along the *x* axis and intensity of signal along the *y* axis. (**E/L**) The ratios between the C and T alleles of SNP rs45454293 (**E**) and the A and G alleles of SNP rs3850641 (**L**) of phosphorylated Pol II loading compared with input chromatin used in ChIP reactions are shown. Data are expressed as mean (95% c.i.) of two independent immunoprecipitation reactions, with each immunoprecipitation analyzed by PCR in up to three replicates.

Messenger RNA measurements confirmed that the *TNFSF4* gene was expressed in these cells (Fig. S2), in agreement with a previous report [24]. Considering that both cell types express *TNFSF4*, B cells were used because heterozygous with respect to both polymorphisms unlike the monocytic cell lines we used for EMSAs (see below). There was an allele-specific difference in loading of phosphorylated Pol II to the *TNFSF4* gene (Fig. 2A–E). The mean C:T ratio was 1.13 (95% confidence interval (c.i.) = 0.77–1.49) after immunoprecipitation compared with 1 (95% c.i. =  0.56–1.44) in the starting material (total input chromatin), however the difference was not significant (P = 0.46). Fragments bound to phosphorylated Pol II contained predominantly the rs3850641 A-allele (P = 0.02), with an A:G ratio of 1.38 (95% c.i. =  1.10–1.66) compared with 1 (95% c.i. =  0.74–1.26) for the input chromatin (Fig. 2F–L). The corollary of these findings is that *in vivo* the haplotype containing the rs45454293T and the rs3850641G alleles is associated with lower transcriptional activity.

### The rs45454293T allele reduces gene expression

**Figure 3:**
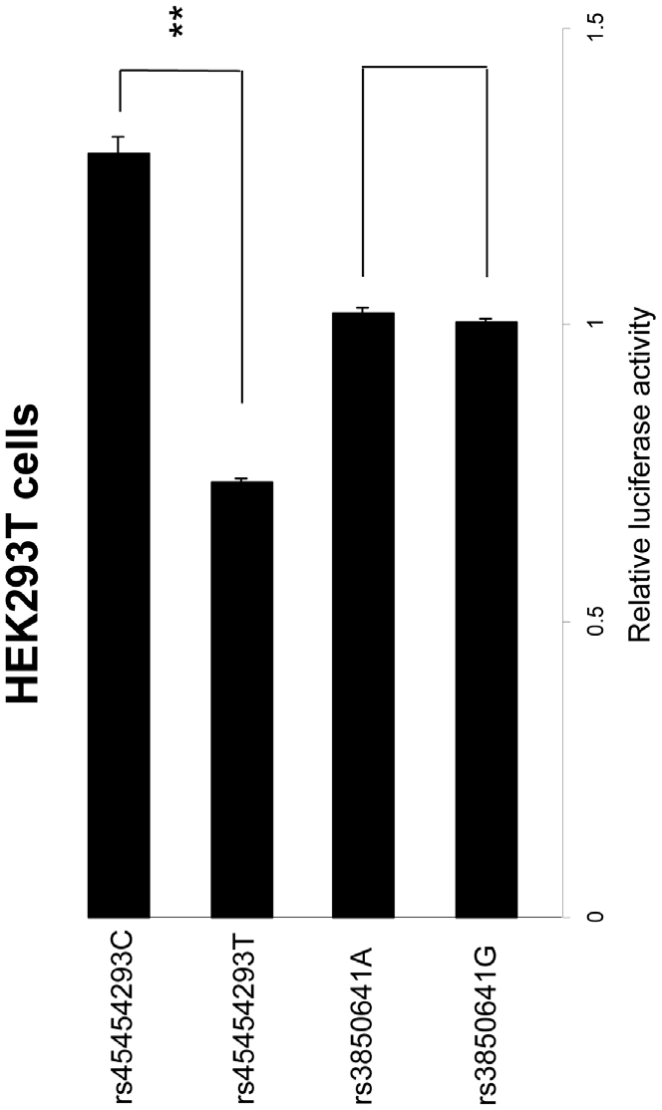
Transcriptional regulatory activity on *TNFSF4* polymorphisms in HEK293T cells. Relative activity was calculated by taking the relative luciferase activity of the empty vector to be 1. Data show the relative activity (mean ± s.e.m) from three experiments done in duplicate. *P = 0.0005.

To clarify which of the two polymorphisms included in the TG haplotype had a functional role, cells from the HEK293T line were transiently transfected with sequences carrying either allelic variant for both SNPs. A clone containing the rs45454293T allele showed a reduction in transcriptional activity of almost 50% compared with the rs45454293C allele (0.73 vs 1.29, P = 0.0005; Fig. 3); in contrast, there was no significant difference when cells were transfected with the rs45454293C allele or the empty vector (1.29 vs 1, P = 0.08; Fig. 3). No difference was observed when cells were transfected with clones containing either allelic variant for rs3850641 (P = 0.42), values being comparable also to cells transfected with the empty vector alone (P = 0.24).

### Allele-specific binding of nuclear proteins to the polymorphic sites

**Figure 4:**
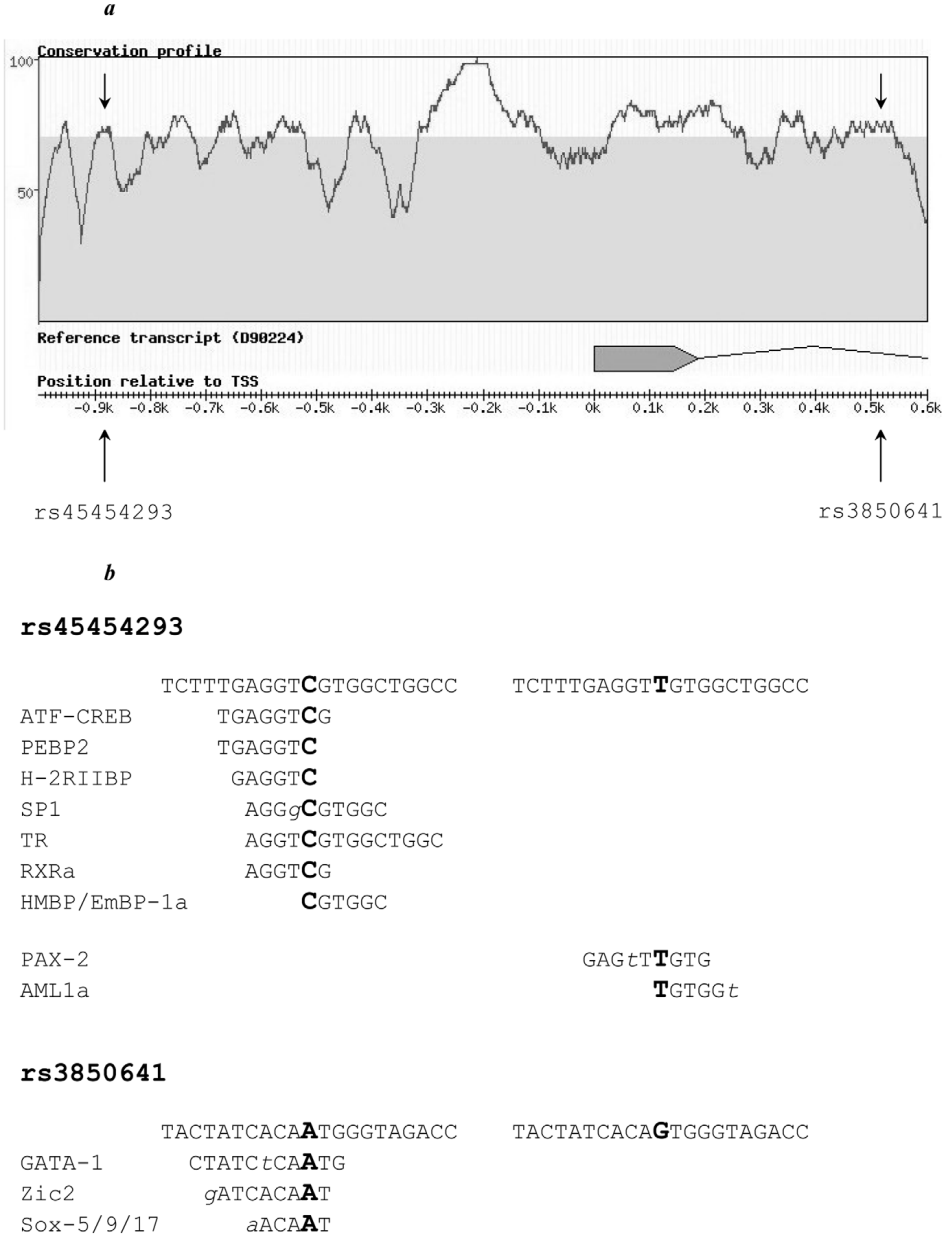
(A) *TNFSF4* region across the transcription start site. Conservation profile between human and mouse obtained using the RAVEN software. Area above the gray area indicates a degree of homology >70%; arrows represent tested SNPs. The *TNFSF4* gene was retrieved from the NCBI database (GenBank accession number D90224). (**B**) **Sequence of the** ***TNFSF4*** **promoter between positions –931 and –911 (upper) and of intron 1 between positions +474 and +494 (lower), indicating the polymorphic sites.** Potential binding sites for different transcription factors are indicated for both alleles. Allelic variants for each polymorphism are indicated in bold, mismatches between *TNFSF4* sequence and transcription factor binding sites are indicated in lower case italic.

Phylogenetic footprinting analysis using the RAVEN software showed that the rs45454293 and rs3850641 polymorphisms are located in regions that are highly conserved between mouse and man, indicating that these regions may be of functional importance (Fig. 4A).

A thorough analysis of the region that harbours both polymorphisms of interest to look for regulatory elements (histone modifications, presence of transcription factor binding sites, evidence of open chromatin and existence of DNaseI hypersensitive sites) within the ENCODE project was performed in cell lines relevant to *TNFSF4* pattern of expression i.e. lymphoblastoid lines (including Jurkat), B and T lymphocytes and HUVEC. It revealed that potential regulatory elements are located mostly elsewhere upstream of the TSS in the large intron 2. Though, intronic SNP rs3850641, located at position 173175832, co-localises with a region associated with histone modifications (H3K36me3: Histone H3 (tri-methyl K36)) between 173175736 and 173175930 bp, while promoter SNP rs45454293, located at position 173177236, is located just a few base pairs away from two regions also associated with histone modifications (CTCF zinc finger transcription factor) between 173177077 and 173177207 bp, and 173177297 and 173177323 bp. In addition to these locations several other regions were found that are predicted to include transcription factor binding sites and DNaseI hypersensitive sites, which map approximately 100 to 1000 bp away from the rs45454293 and rs3850641, but it is not clear to what extent the two SNPs can affect binding of these regulatory factors.

**Figure 5:**
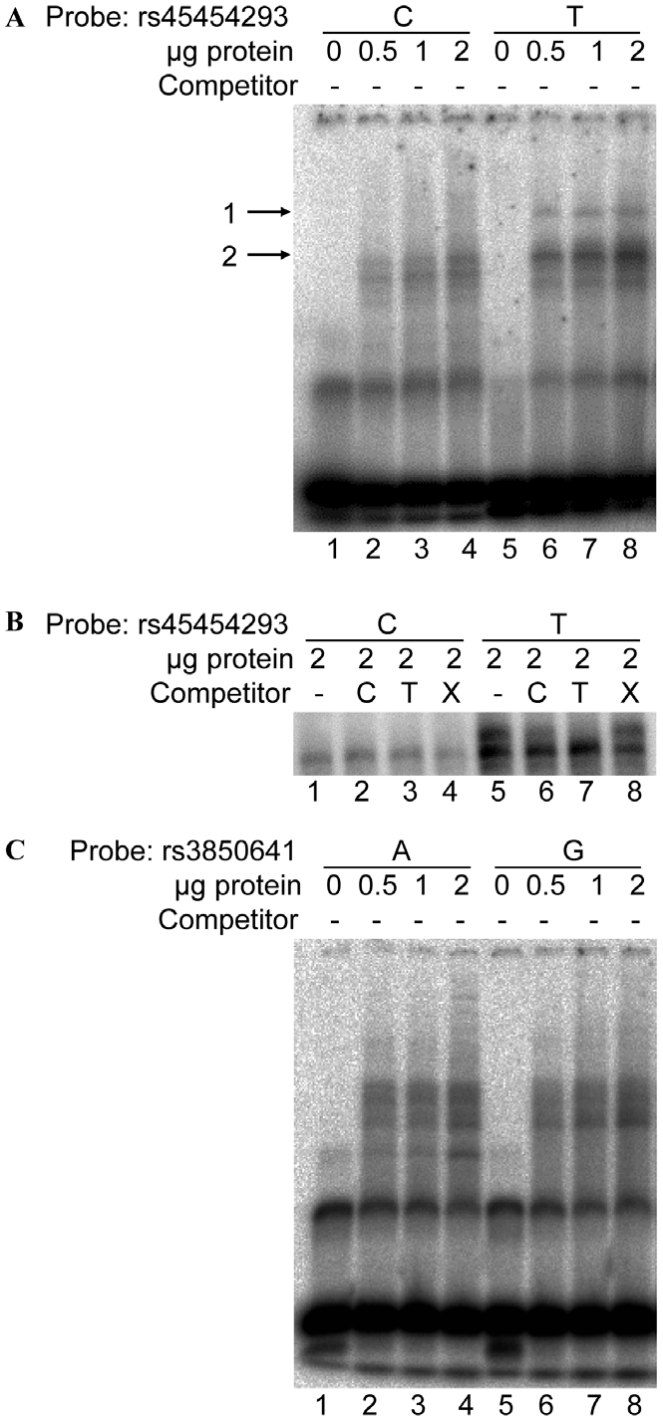
Representative EMSAs of nuclear extract derived from U937 cells bound to the rs45454293 region and the rs3850641 region. (**A**) EMSA of a 25 bp DNA fragment containing either the rs45454293C (lanes 1-4) or the rs45454293T site (lanes 5-8). Lanes 1 and 5, no extract; lanes 2 and 6, 0.5 µg of extract; lanes 3 and 7, 1 µg of extract; lanes 4 and 8, 2 µg of extract. Arrow denotes the specific DNA-protein complexes associated with the polymorphic sites. (**B**) Competition experiment demonstrating specific binding to the rs45454293T allele: lanes 1 and 5, without competitors; lanes 2 and 6, rs45454293C and rs45454293T probe, respectively, with a 100-fold excess of rs45454293C probe as competitor; lanes 3 and 7, rs45454293C and rs45454293T probe, respectively, with a 100-fold excess of rs45454293T probe as competitor; lanes 4 and 8, rs45454293C and rs45454293T probe, respectively, with a 100-fold excess of non-specific (X) competitor. (**C**) EMSA of a 25 bp DNA fragment containing either the rs3850641A (lanes 1–4) or the rs3850641G site (lanes 5-8).

The possibility that the rs45454293 and/or rs3850641 SNPs affect the binding of nuclear proteins was analyzed by EMSAs. Allele-specific binding of nuclear proteins was observed when nuclear extracts derived from U937 cells were incubated with oligonucleotide probes spanning the rs45454293 polymorphism (Fig. 5A,B). The rs45454293T-allele induced a protein-DNA complex that was not present with the rs45454293C-allele (Fig. 5A,B). A similar allele-specific binding pattern was obtained using nuclear extract derived from human THP-1 cells (data not shown). In contrast, there was no allele-specific binding to the rs3850641 polymorphism using nuclear extracts derived from U937 cells (Fig. 5C).

*In silico* analysis using the TRANSFAC database indicated that the rs45454293T-allele induces a binding site for the transcription factor AML1a, a protein affecting granulocyte differentiation and proliferation (Fig. 4B). In addition, the rs45454293T-allele creates a binding site for the transcription factor PAX-2, a factor involved in development of renal epithelium by induction of tumor suppressor genes [25] as well as in cell proliferation and carcinogenesis [26]. However, EMSAs including antibodies against these transcription factors did not demonstrate a supershift of the T-specific complex (data not shown).

## Discussion

The TNFSF4/TNFRSF4 system, along with several other receptor-ligand pairs, has been suggested to be involved in the recruitment and activation of T-cells and is therefore tentatively implicated in atherosclerosis and acute coronary syndromes such as MI. We have previously demonstrated that a *TNFSF4* haplotype is associated with risk of MI in women and that genetic variants in the human *TNFSF4* gene are associated with similar intermediate phenotypes to the ones associated with the *Ath1* locus in mice [5]. In the present study, we searched for functional SNPs and haplotypes contained in the *TNFSF4* gene. The rs45454293 promoter polymorphism was shown to conceivably influence gene regulation and to account for the previously described association between a *TNFSF4* haplotype and MI.

In order to dissect the mechanism behind the observed association between *TNFSF4* haplotypes and MI, and to identify the polymorphism(s) responsible for the perturbation of gene expression/activity, we used the haploChIP method to investigate whether the putative regulatory rs3850641 and rs45454293 SNPs influence Pol II loading, an indirect measure of allele-specific gene expression *in vivo* in the presence of a natural chromatin structure. We selected these two specific SNPs for functional analyses because they were the only ones found to be associated with MI. Differences between the two alleles were observed for both SNPs (ratio of 1.13 and 1.38), indicating that the functional significance resides in the haplotype defined by these polymorphisms. Specifically, the haplotype carrying the T-allele of the rs45454293 SNP and the G-allele of the rs3850641 SNP was associated with decreased loading of activated polymerase II, i.e. with lower transcriptional activity. Needless to say, the effect is small and this result is only based on EBV transformed B-cells and should therefore be interpreted with caution. However, the results of the transient transfection studies in HEK293T cells provide further support for a functional role of the rs45454293 polymorphism. It should be emphasized in this context that the lack of endogenous promoter elements for rs3850641 may influence the results for this SNP, as transcription might be affected by interaction of transcription factors binding at various sites of the promoter. Nevertheless, the results obtained allow us to determine which of the two minor alleles (rs45454293T or rs3850641G) indicated by the haploChIP experiment to be associated with lower transcriptional activity, has a functional effect on transcription.

An important question is whether the apparently small variation in gene activity, ranging between 10% and 40%, can have a physiologically significant effect. Importantly, the extent of this effect can be more substantial considering that measurements were performed under basal conditions, i.e. in cells that were not stimulated. Indeed, the gender-specific effect observed suggests that there might be environmental effects, including sex hormones, amplifying the allele/haplotype-specific differences in transcriptional activity. In fact, the importance of such small variation is indicated by the transfection studies which confirm that the risk T-allele of the rs45454293 polymorphism negatively affects transcriptional regulation, providing a mechanistic insight for the observed allele-specific decrease in *TNFSF4* expression.

The rs45454293T-allele was shown to be associated with MI in women, thus suggesting that lowered *TNFSF4* expression is associated with increased risk of MI. The role of this SNP in inflammation and thromboembolic disease was suggested by a study of Malarstig et al. [27] where the rs45454293T-allele was associated with an increased risk of venous thromboembolism in women but not in men, reinforcing the hypothesis that carriers of this allele have increased systemic inflammation and therefore might be particularly prone to plaque rupture. The rs45454293 polymorphism, along with the previously described rs3850641 SNP [5], was also demonstrated to be part of a haplotype significantly associated with MI in the female subset of the cohort. Our experimental evidence suggests that the minor allele of rs45454293 rather than the rs3850641 polymorphism is responsible for the haplotype-MI association. Both these findings are in line with the original results that genetic variation in *TNFSF4* is associated with inflammatory markers and MI in women in two independent cohorts [5], suggesting a gender-specific effect on gene regulation. Of note, the gender-specific effect of *TNFSF4* is also present in mice, female mice being more susceptible to atherosclerosis than male mice [4]. Nevertheless, further molecular and large-scale association studies are needed to confirm the gender-specific effect, also in relation to *TNFSF4* genetic variation.

It is notable that the rs3850641 SNP did not influence the binding of nuclear proteins as shown by our EMSA studies. In contrast, these experiments suggested that the rs45454293 polymorphism affects the binding of nuclear factors, protein complexes showing differential binding to the rs45454293T-allele. Taken together, the results of the haploChIP, transient transfections and EMSA studies suggest that rs45454293 is the functional polymorphism and that the lower transcriptional activity associated with the rs45454293T-allele is due to binding of one or more transcriptional repressor(s) to the T-allele.

Since the two *TNFSF4* SNPs examined in the present study have not come up as genome-wide significant in the hitherto published genome-wide association studies (GWAS) for MI, it is likely that *TNFSF4* does not belong to the group of major coronary artery disease susceptibility genes that survive the fairly conservative adjustments for multiple-testing applied on the hypothesis-free, high-density, high-coverage SNP genotyping in GWAS. However, the region containing the *TNFSF4* gene has been found to be associated with celiac disease, a chronic inflammatory disease with a strong immune component [28] and systemic lupus erythematosus, an autoimmune disease [29], which are likely to share common inflammatory pathways with atherosclerosis, the main underlying cause of MI.

We have previously shown that *Tnfsf4* contributes to atheroma formation using both knockout and transgenic mice [5], and it is notable in this context that lesions in these strains are similar to early fatty streaks in humans, consisting primarily of foam cells without smooth muscle cells (SMCs) and fibrous cap formation [30]. Furthermore, the mouse strains carried genetic variations in the *Tnfsf4* promoter region that affected gene activity [5], as shown here for humans. However, unlike human plaques, the plaques found in the atherosclerosis-susceptible mice are not prone to rupture. Thus, it appears that harbouring this specific genetic variation in *TNFSF4* promotes a pro-inflammatory state in humans that destabilizes the atherosclerotic plaque, making it particularly prone to rupture. For as yet unknown reasons, this effect seems to be gender-specific, being confined to women.

The fact that TNFSF4 is expressed by several cell types suggests that TNFSF4 has more functions than the originally reported involvement in T-cell activation [1], [31]. Of cells present in the atherosclerotic lesion, both endothelial cells, macrophages, mast cells and SMCs express TNFSF4 [3]. Therefore, the observed genotype-phenotype associations could reflect the net effect of TNFSF4 actions in different cell types. Expression of TNFSF4 on different types of antigen-presenting cells (macrophages, dendritic cells, B cells and SMCs) might influence T-cell recognition of antigens, such as altered epitopes on oxidatively modified LDL particles. In addition, TNFSF4 expressed on mast cells may interact with TNFRSF4 on T-cells and stimulate their proliferation [32]. The cross-talk between the two cell types might exert an effect also in the opposite direction, regulating mast cells and their role in inflammation, as has been observed for other members of the TNF/TNF receptor (TNFR) superfamily. In fact, mast cells can be activated by T-cell-dependent co-stimulatory signals transduced by ligation of lymphotoxin-β [33] and 4-1BBL [34]. Finally, TNFSF4 expressed on endothelial cells was reported to mediate the adhesion of TNFRSF4-expressing T-cells to vascular endothelial cells and the subsequent migration to distant inflammatory sites [35], suggesting an involvement of TNFSF4 in lymphocyte recruitment as well. Unstable plaques are particularly rich in activated lymphocytes [36]; therefore all these events, possibly triggered by TNFSF4, may favor destabilization and rupture of the plaque.

In conclusion, the present work suggests that lowered *TNFSF4* expression is associated with an increased risk of MI. Further analyses are now needed to precisely determine the function of the TNFSF4 protein in MI. Specifically, the gender difference needs to be evaluated on a molecular level.

## Supporting Information

Figure S1 **Representation of degree of linkage disequlibrium, in terms of D′ and LOD scores, across the** ***TNFSF4*** **gene based on 9 variants genotyped in the SCARF cohort.** Each square is generated by the intersection between two SNPs and indicates the corresponding D′ value; empty square means D′ = 1.
(TIF)

Figure S2 **Expression levels of** ***TNFSF4*** **in EBV-transformed human B cell lines.** Standards are depicted in blue and Ct values from real-time RT-PCR are presented for *TNFSF4* expression when cells were kept at basal conditions and upon various stimuli (IL-1b: Interleukin-1 beta, TNFa: Tumor Necrosis Factor-alpha, IFNg: Interferon-gamma).
(TIF)

Table S1 **PCR primers.**
(DOC)

Table S2 **Nested sequencing primers.**
(DOC)

Table S3 **Pyrosequencing primers.**
(DOC)

Table S4 **Genotype distributions and Hardy-Weinberg equilibrium.**
(DOC)

Table S5 **Distribution of TNFSF4 haplotypes in patients and control subjects (764 individuals/1528 alleles).**
(DOC)
