# Supplementary figures and images for: A Common Polymorphism in the Promoter Region of the TNFSF4 Gene Is Associated with Lower Allele-Specific Expression and Risk of Myocardial Infarction
Source: PLoS One. 2011 Mar 18;6(3):e17652. doi: 10.1371/journal.pone.0017652 (PMC3060868; doi:10.1371/journal.pone.0017652)

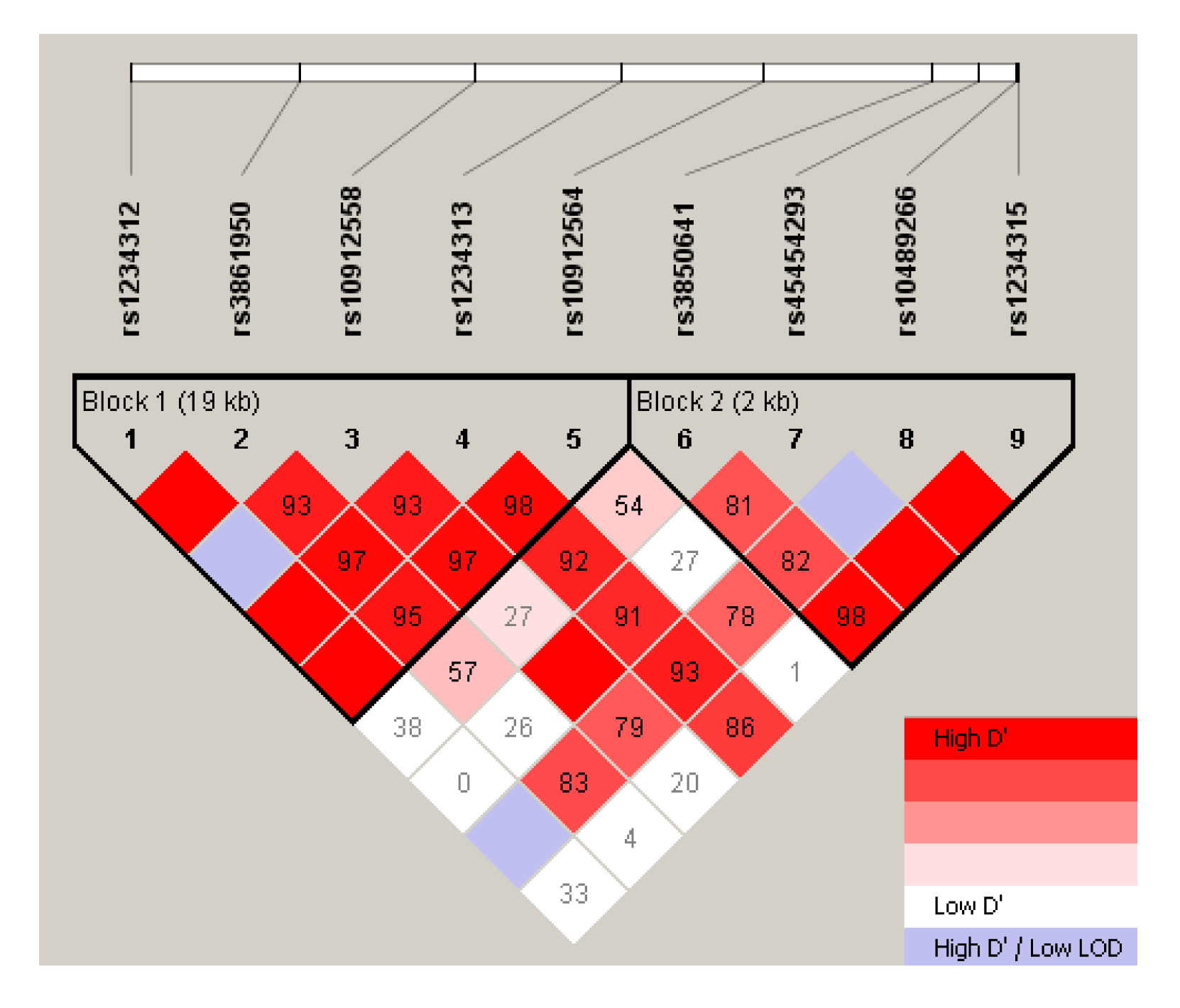

Supplement: Figure S1 — Representation of degree of linkage disequlibrium, in terms of D′ and LOD scores, across the TNFSF4 gene based on 9 variants genotyped in the SCARF cohort. Each square is generated by the intersection between two SNPs and indicates the corresponding D′ value; empty square means D′ = 1. (TIF) [file pone.0017652.s001.tif]

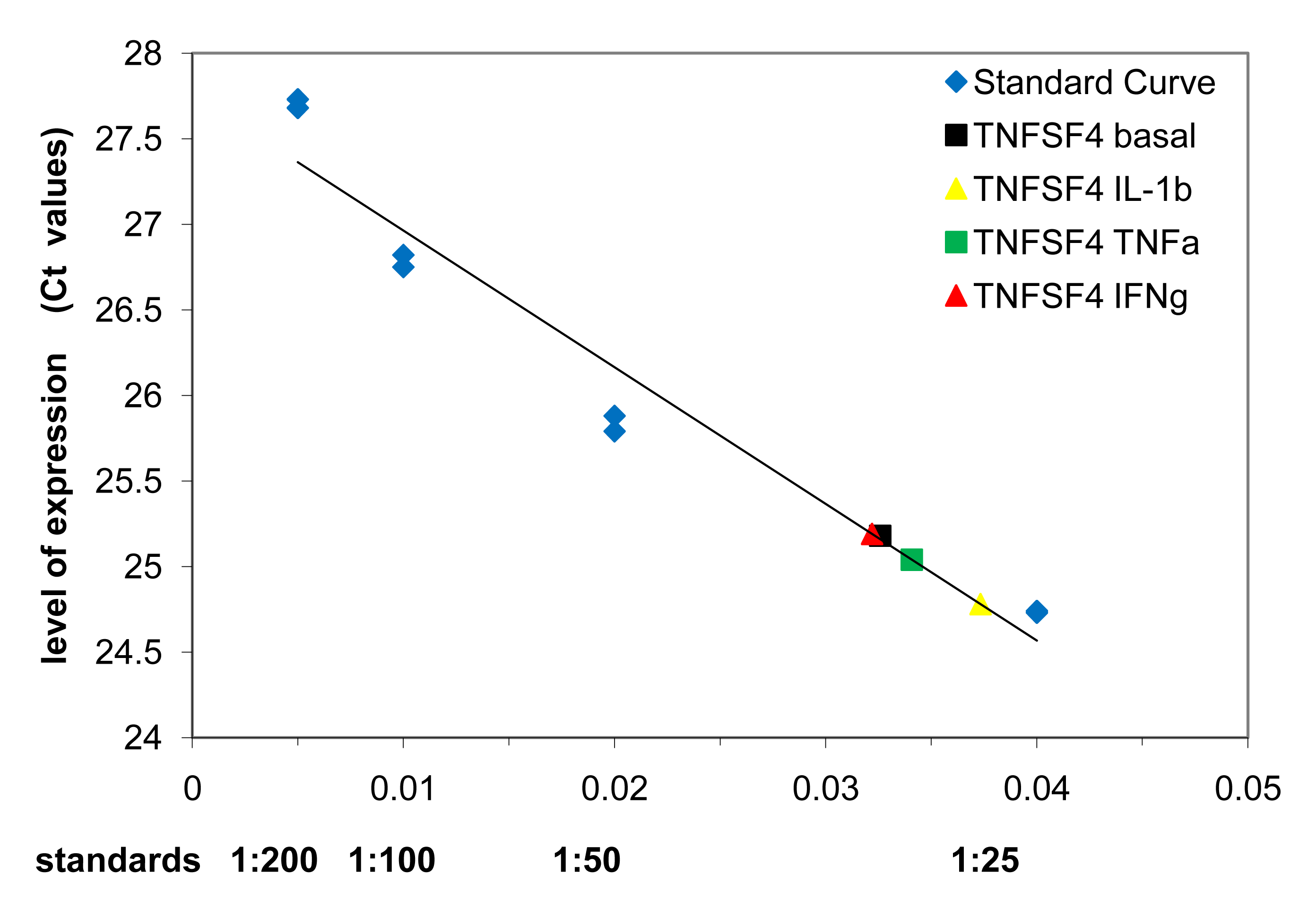

Supplement: Figure S2 — Expression levels of TNFSF4 in EBV-transformed human B cell lines. Standards are depicted in blue and Ct values from real-time RT-PCR are presented for TNFSF4 expression when cells were kept at basal conditions and upon various stimuli (IL-1b: Interleukin-1 beta, TNFa: Tumor Necrosis Factor-alpha, IFNg: Interferon-gamma). (TIF) [file pone.0017652.s002.tif]
