# Supplementary material for: A Common Polymorphism in the Promoter Region of the TNFSF4 Gene Is Associated with Lower Allele-Specific Expression and Risk of Myocardial Infarction
Source: PLoS One. 2011 Mar 18;6(3):e17652. doi: 10.1371/journal.pone.0017652 (PMC3060868; doi:10.1371/journal.pone.0017652)
Supplement: Table S1 — PCR primers. (DOC) [file pone.0017652.s003.doc]

**Table S1. PCR primers**

| Primer name | Primer sequence (5’ to 3’) |
| --- | --- |
| -2449F* | AGGCTGAGGCAGGAGGAT |
| -1108R* | GGGGAAAAGCTGTTTAAGACG |
| -2055F* | TGAAGAGCTGTGAAAACGTG |
| -486R* | TGGCCCAAGGTTCTTATCTG |
| -733F* | TGAGAGAAAGATTGCAAGTCCA |
| 374R* | TCTGCCCTACCTCCACTCTG |
| 3625F | AATAAGAGAAAGAGGAGTTTGCCTG |
| 4201R | CATCTTCCCATTGTCCCTTGAG |
| 5515F | GGCCAGGAACCAGAATTTTT |
| 6409R | GAAGTGAGGGCTCCAGTGAG |
| 14214F | GTTTTCCGTGTGCATCCAAG |
| 14646 | AAGATCAGTGGTGCCAGGG |
| 23298F | TATACTGGGCAGGTCCCCTA |
| 24080R | CTAATACGATGCCTGGCACA |

* used also as sequencing primer.
