# Supplementary material for: A Common Polymorphism in the Promoter Region of the TNFSF4 Gene Is Associated with Lower Allele-Specific Expression and Risk of Myocardial Infarction
Source: PLoS One. 2011 Mar 18;6(3):e17652. doi: 10.1371/journal.pone.0017652 (PMC3060868; doi:10.1371/journal.pone.0017652)
Supplement: Table S2 — Nested sequencing primers. (DOC) [file pone.0017652.s004.doc]

**Supplementary Table 2. Nested sequencing primers**

| Primer name | Primer sequence (5’ to 3’) |
| --- | --- |
| -1177R | GGCACCCAGCAGATATGTAAA |
| -1587F | AATTGGGCTTTACACACTG |
| -733F | TGAGAGAAAGATTGCAAGTCCA |
| -1561R | GGAAAAATCAGTGTGTAAAGCC |
| 3943F | GGGTAGGTGAACCATTCGTG |
| 4180R | GCATGTCACTCTTTTGTCCCCA |
| 5531F | TTTTCAGTCCAGGCTTTTACCTC |
| 5976F | TGTCTGCACGTGGTTCAGAGA |
| 14386F | ATGCAATTTTCCCAAGGTCA |
| 14412R | AGCTGTGTGACCTTGGGAAA |
| 23909R | AAAGCTGGGGAACAGAAGTG |
| 23751F | TGTCCCCTCTCCTAAGTGGT |
