# Supplementary material for: A Common Polymorphism in the Promoter Region of the TNFSF4 Gene Is Associated with Lower Allele-Specific Expression and Risk of Myocardial Infarction
Source: PLoS One. 2011 Mar 18;6(3):e17652. doi: 10.1371/journal.pone.0017652 (PMC3060868; doi:10.1371/journal.pone.0017652)
Supplement: Table S3 — Pyrosequencing primers. (DOC) [file pone.0017652.s005.doc]

**Supplementary Table 3. Pyrosequencing primers**

| Primer name | Primer sequence (5’ to 3’) |
| --- | --- |
| -921 for | CAGAAAAGAAGCAGAAGGGTATCC |
| -921 rev | TGGTAAAGGGTACCTGGTGTCTAT |
| -921seq | ATTATTTCTTTCTTTGAGGT |
| rs3850641 for | AAAATCACCTTGCAGGTCTATAGGG |
| rs3850641 rev | TGCAATGCAAGATTCCTTGT |
| rs3850641 seq | TCAAACACATTACTATCACA |
