# Supplementary material for: A Common Polymorphism in the Promoter Region of the TNFSF4 Gene Is Associated with Lower Allele-Specific Expression and Risk of Myocardial Infarction
Source: PLoS One. 2011 Mar 18;6(3):e17652. doi: 10.1371/journal.pone.0017652 (PMC3060868; doi:10.1371/journal.pone.0017652)
Supplement: Table S4 — Genotype distributions and Hardy-Weinberg equilibrium. (DOC) [file pone.0017652.s006.doc]

**Supplementary Table 4. Genotype distributions and Hardy-Weinberg equilibrium.**

| Polymorphism | Genotype | Observed | Expected under HWE | P value |
| --- | --- | --- | --- | --- |
| rs10489266 | AA | 632 | 634 | 0.36 |
|  | AG | 128 | 124 |  |
|  | GG | 4 | 6 |  |
| rs45454293 | CC | 665 | 663.5 | 0.40 |
|  | CT | 94 | 97 |  |
|  | TT | 5 | 3.5 |  |
| rs10912564 | GG | 345 | 346.5 | 0.81 |
|  | GA | 337 | 334 |  |
|  | AA | 79 | 80.5 |  |
| rs10912558 | CC | 478 | 485.5 | 0.10 |
|  | CA | 262 | 247 |  |
|  | AA | 24 | 31.5 |  |

If P > 0.05 the population is in Hardy-Weinberg equilibrium.
