# Supplementary material for: A Common Polymorphism in the Promoter Region of the TNFSF4 Gene Is Associated with Lower Allele-Specific Expression and Risk of Myocardial Infarction
Source: PLoS One. 2011 Mar 18;6(3):e17652. doi: 10.1371/journal.pone.0017652 (PMC3060868; doi:10.1371/journal.pone.0017652)
Supplement: Table S5 — Distribution of TNFSF4 haplotypes in patients and control subjects (764 individuals/1528 alleles). (DOC) [file pone.0017652.s007.doc]

**Supplementary Table 5. Distribution of *TNFSF4* haplotypes in patients and control subjects (764 individuals/1528 alleles).**

| Haplotype | Total (%) | Controls (%) | Patients (%) | P value |
| --- | --- | --- | --- | --- |
| 00 | 1302 (85.2) | 673 (87.0) | 629 (83.4) | 0.06 |
| 01 | 120 (5.9) | 53 (6.8) | 67 (8.9) | ns |
| 10 | 16 (1.0) | 8 (1.0) | 8 (1.1) | ns |
| 11 | 90 (5.9) | 40 (5.2) | 50 (6.6) | ns |

P values were calculated using the chi-square test for genotype distribution. 0 = ancestral allele, 1 = minor allele.
